# The interplay between pulsatility, sympathetic regulation and renal physiology: Implications for left ventricular assist devices

**DOI:** 10.1113/EP093780

**Published:** 2026-06-25

**Authors:** Tania Warnakulasuriya, Zaid Bahi, Rohit Ramchandra

**Affiliations:** ^1^ Department of Physiology, Faculty of Medical and Health Sciences University of Auckland Auckland New Zealand; ^2^ Department of Physiology, Faculty of Medicine University of Kelaniya Ragama Sri Lanka

**Keywords:** left ventricular assist device, pulsatility, renal function, sympathetic nerve activity

## Abstract

Left ventricular assist devices have gained traction both as a bridge therapy and as a destination therapy in the management of heart failure. Many of these devices reinstate blood flow in a continuous manner as opposed to the pulsatile flow present in normal physiology. This loss of pulsatility can alter the arterial baroreflex and sympathetic nervous system as well as modify release of nitric oxide. This review focuses on the relationship between pulsatility and sympathetic nerve activity with an emphasis on how this may alter kidney function. Our findings indicate that, in the clinical context of ventricular dysfunction and mechanical circulatory support, pulsatile flow may lower levels of sympathetic nerve activity and theoretically improve nitric oxide function. While there are inhibitory effects on sympathetic nerve activity during pulsatile flow, the downstream effects on renal function remain inconclusive with the caveat that these studies are often underpowered retrospective observational studies that use indirect measures of renal function. We suggest that future studies need to incorporate more sensitive markers of tubular and glomerular function to establish if pulsatility indeed has positive outcomes on renal function. Based on current data, we speculate that the improvement in cardiac output and redistribution of blood flow within the kidney may override putative beneficial effects of altered pulsatility observed in experimental studies.

## Abstract (highlights)

- **What is the topic of this review?**
  This review explores the interactions between pulsatility and sympathetic nerve activity, with an emphasis on implications for renal physiology.
- **What advances does it highlight?**
  Evidence from clinical studies of mechanical circulatory support suggests that restoration of pulsatile flow is associated with reduced sympathetic outflow. Furthermore, pulsatility may also favour nitric oxide bioavailability. Clinical studies examining renal function are inconclusive with the caveat that these studies are often underpowered retrospective studies that use indirect measures. Future studies need to incorporate more sensitive markers of tubular function to determine if pulsatility leads to positive outcomes.

## 1 INTRODUCTION

**FIGURE 1:**
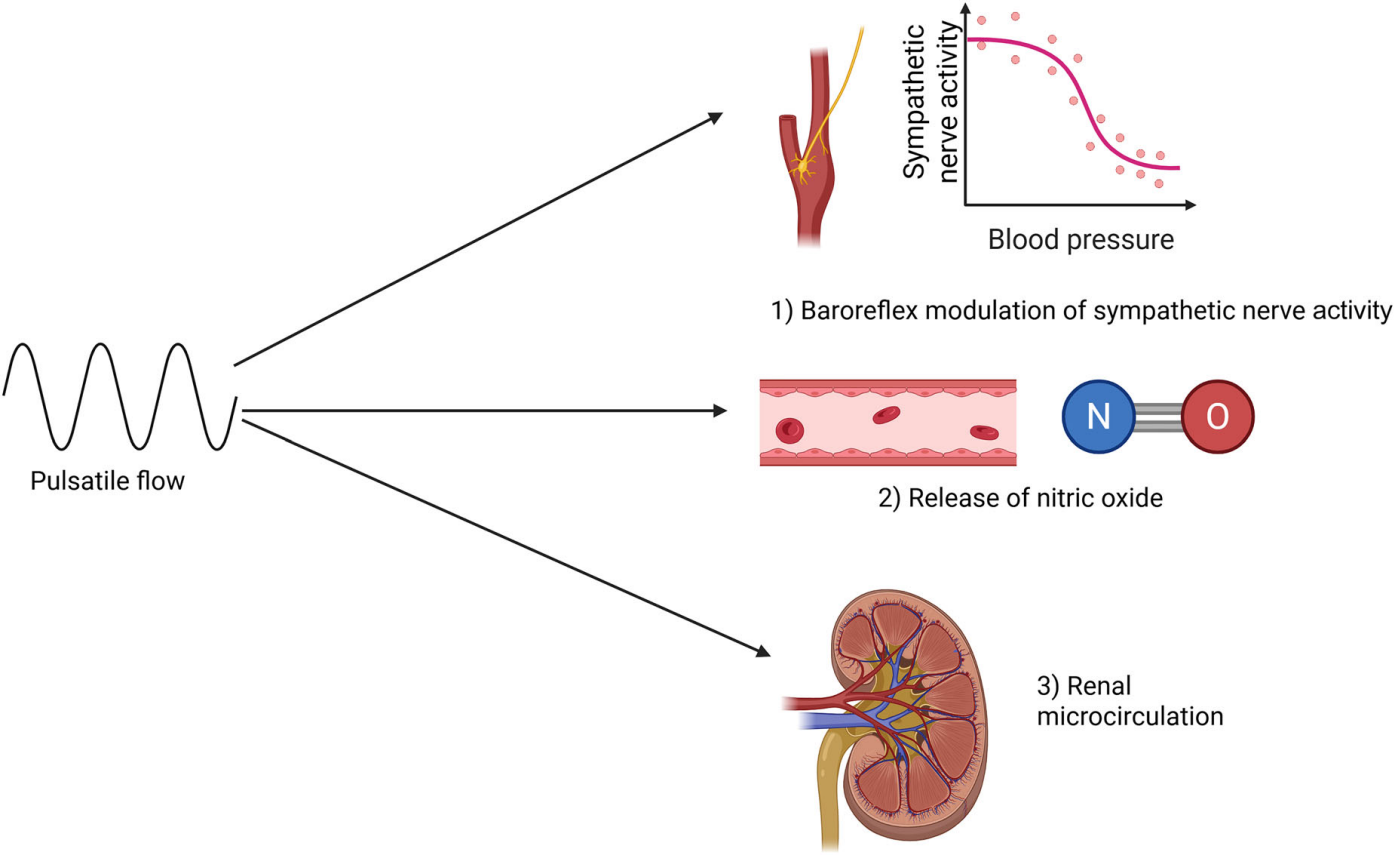
Physiological effects of pulsatile flow. A schematic representation highlighting how the physiological effects of pulsatile flow can be conceptualized as operating through three interconnected pathways: (1) baroreflex‐mediated modulation of sympathetic nerve activity, (2) endothelial responses to pulsatile shear stress that influence nitric oxide bioavailability, and (3) downstream regulation of renal microvascular perfusion.

Globally, cardiovascular disease remains a major cause of premature mortality and rising health care burden (Roth et al., 2020) with heart failure (HF) often following ischaemic heart disease and contributing to mortality. Left ventricular assist devices (LVADs) have gained traction as both a bridge and a destination therapy in the management of both chronic and acute HF (Heidenreich et al., 2022). Today, there are many types of LVADs available for clinical use, the majority of which reinstate blood flow in a continuous manner as opposed to the pulsatile flow present in normal physiology. Whether the loss of pulsatility has downstream effects on morbidity and end‐organ function has been reviewed in the past (Miller, 2009) with no clear consensus. One system that is altered by loss of pulsatility is the sympathetic nervous system, and one aim of this review is to focus on the relationship between sympathetic drive and pulsatility. Why is this important? The sympathetic nervous system is mechanistically linked to both acute and chronic complications in HF. Modulation of elevated sympathetic nerve activity (SNA) has been a key goal in the management of HF to improve patient outcomes. Whether the reduction in pulsatility compromises end‐organ function via elevations in SNA or through direct effects on the vasculature is an important research question in relation to LVAD therapy in HF. The physiological effects of pulsatile flow can be conceptualized as operating through three interconnected pathways: (1) baroreflex‐mediated modulation of SNA, (2) endothelial responses to pulsatile shear stress that influence nitric oxide (NO) bioavailability, and (3) downstream regulation of renal microvascular perfusion (Figure 1). These pathways likely interact to influence renal haemodynamics in patients supported with LVADs. In the following sections, we discuss each mechanism individually before integrating them into a unified framework linking pulsatility to renal outcomes.

In this review, a targeted literature search was used to capture key studies relevant to the physiological and clinical effects of flow pulsatility. Specifically, three databases were used for the literature search: PubMed, MEDLINE and Scopus. All of these databases were used to search for publications from inception to August 2025. A combination of keywords was used to perform the literature search, including: ‘LVADs’, ‘Renal Function’, ‘Continuous Flow’, ‘GFR’, ‘renal perfusion’ and ‘Pulsatility’. The output identified studies reporting renal function in patients with continuous and/or pulsatile flow LVADs. Following identification of relevant literature, publication eligibility was determined based on whether they used a LVAD, reported renal function measures, and were experimental, prospective or retrospective studies. Studies that did not specify device type or did not report renal function markers were excluded. All studies included were limited to those in English text and involving animal or human subjects/models. We acknowledge that narrative reviews are inherently selective and may be subject to bias. To mitigate this, we aimed to include both seminal studies and recent investigations, with a particular focus on mechanistic insights and physiological relevance. No generative AI tools were used in the preparation of this article.

Ejection of blood from the ventricle during the systolic phase of the cardiac cycle into the aorta and the arteries causes an increase in systolic pressure contributing to the pulse pressure. This also generates the hydrostatic pressure that allows blood to flow from the aorta to the periphery according to the pressure gradient (Briet et al., 2012; Molnar & Gair, 2015). The downward stroke of the pulse occurs during ventricular diastole. Multiple physiological mechanisms have evolved/adapted to detect and respond to pulsatile flow in the arterial circulation. These mechanisms have the ability to alter tissue perfusion with loss of pulsatility. We will first focus on a couple of these, the baroreflex feedback arc and release of NO by the endothelial cells (Safar et al., 2001).

### 1.1 Baroreflex‐mediated modulation of sympathetic nerve activity

In this section, we focus on the mechanisms whereby pressures are sensed by the baroreceptors of the cardiovascular system to understand how pulsatility can influence haemodynamics. The high‐pressure baroreceptors are located in the carotid sinus and the aortic arch (Glick & Covell, 1968). Stretch of these baroreceptors leads to activation of mechanosensory channels in nerve endings, including piezo channels, stretch activated potassium channels, acid sensing ion channels (Lu et al., 2009) and transient receptor potential channels (Fang et al., 2021), which leads to depolarization of afferent neurons. An increase in baroreceptor afferent signals is relayed specifically to the nucleus tractus solitarius in the medulla. Second‐order neurons from the nucleus tractus solitarius synapse with neurons in the caudal ventrolateral medulla, a key depressor of the vasomotor area (Blessing & Li, 1989; Drolet et al., 1993). These caudal ventrolateral medulla neurons send inhibitory projections to the rostral ventrolateral medulla which is a key pressor area (Agarwal et al., 1990) where most of the premotor vasoconstrictor sympathetic neurons arise from (Kumada et al., 1979). Release of inhibition of neurons in the rostral ventrolateral medulla increases SNA, which can then cause an increase in arterial pressure (Angell James, 1971; Angell James & Daly, 1971; Kezdi & Geller, 1968). The nucleus tractus solitarius neurons also project to cardiac vagal neurons in the nucleus ambiguous to decrease cardiac vagal drive (Morrison & Cao, 2000). The compensatory changes in arterial pressure are mediated by concurrent efferent activity to the heart, blood vessels, the kidney and adrenal glands.

## 2 CONSEQUENCES OF ALTERED PULSATILITY ON THE BAROREFLEX

During pulsatile flow, activity in the baroreceptor afferent nerves is silent during low diastolic pressures (Angell James, 1971). Compared with continuous flow, pulsatile flow lowers the threshold for activation of the baroreceptor afferent nerves (Ead et al., 1952). This effectively means that when the pulse pressure declines, the threshold blood pressure at which the baro‐afferent neurones are recruited decreases. Consequently, during steady non‐pulsatile flow, the blood pressure threshold for afferent nerve activation is lower than normal (Angell James, 1971) and should theoretically result in a higher SNA compared to pulsatile flow with a comparable mean arterial pressure.

Experimentally, Angell James (1971) recorded the frequency of impulses in a single baro‐afferent fibre recording during pulsatile and non‐pulsatile flow and found these to be the same during the two scenarios. However, during pulsatile flow, the higher amplitude of nerve activity (multifibre afferent activity) together with the phasic nature of baroreceptor afferent discharges (Chapleau et al., 1989) is thought to enhance baroreceptor signalling and suppress the medullary vasomotor centre leading to lower SNA.

Increasing the pulse pressure also shifts the carotid–aortic perfusion pressure at which maximal baroreflex sensitivity occurs towards lower values (Angell James & Daly, 1971). Hence, pulsatile flow more effectively inhibits SNA at a given arterial pressure. Changes in pulse pressure that modulate sympathetic efferent nerve activity in turn alter systemic vascular resistance. Given this, Angell James and Daly emphasized the importance of maintaining the pulse pressure as baroreceptor mediated reflex vasoconstriction during non‐pulsatile flow may reduce organ perfusion at an equivalent mean arterial pressure (Angell James & Daly, 1970).

## 3 THE DETRIMENTAL EFFECTS OF ELEVATED SYMPATHETIC NERVE ACTIVITY

Data from the previous section indicate that continuous flow may elevate levels of SNA. Previous studies have examined the consequences of this elevation in SNA during HF. Elevated SNA to the heart following an ischaemic insult to the heart may acutely cause an increase in the cardiac output and improve tissue perfusion. However, chronic β‐ and α‐receptor activation causes cardiotoxicity, worsening of ischaemia and abnormal Ca^2+^ handling by the myocyte (Lompré et al., 2010; Schwartz & Stone, 1977; Zeiher et al., 1989). The elevated noradrenaline concentration in the myocardium causes apoptosis and paves the way for remodelling of the cardiac muscle (Colucci et al., 2000; Nakayama et al., 2007; Yoo et al., 2009). As a downstream phenomenon, sustained and elevated sympathetic activation results in desensitization and downregulation of β‐receptors in cardiac tissues. In addition, the excess Ca^2+^ ions in myocytes cause a pro‐arrhythmogenic state and have been attributed to sudden cardiac death (Schömig et al., 1994; Sipido et al., 2007).

In addition to cardiac SNA, elevation in renal SNA is well documented (Hasking et al., 1986), and the level of renal SNA correlates with the severity of ventricular dysfunction (Rundqvist et al., 1997). Molecular changes in renal medullary nephrons brought on by stimulation of adrenergic receptors, such as increased expression of the Na^+^–K^+^–2Cl^−^ channels (in thick ascending loop) (Plato, 2001) and ENaC (in collecting ducts) (Nozawa et al., 2002), cause Na^+^ retention. This in turn propagates systemic circulatory volume overload. Overall, elevated SNA in HF with reduced ejection fraction is ubiquitous and is associated with poor prognosis (Barretto et al., 2009; Petersson et al., 2005). Taken together these studies indicate that if the mode of pulsatility leads to elevated SNA, this would have deleterious downstream consequences.

## 4 WHAT HAPPENS TO SYMPATHETIC NERVE ACTIVITY IN PATIENTS ON LVADS?

There has been much interest in determining changes to the level of sympathetic output among patients receiving LVADs. The gold‐standard methods used to assess SNA have been direct recordings of muscle SNA and plasma catecholamine measurements. It must be noted that while muscle SNA is a direct measure of sympathetic drive, nerve activity to different organs can be differentially controlled (Malpas, 2010; Ramchandra et al., 2006). This does mean that changes in muscle SNA may not always mean similar changes in SNA to the heart or kidney. For this review, we will primarily focus on studies that have used gold standard direct recordings of muscle SNA in patients with LVADs.

Sailer et al. (2021) reported that in a prospective study with severe HF patients (*n* = 10), plasma noradrenaline levels were reduced significantly with continuous flow LVADs (Heartmate III and Heartware VAD) but remained higher than normal. Similar findings have been observed with pulsatile LVADs (Heartmate) as well (K. B. James et al., 1995). Looking at organ‐specific sympathetic drive, Tank et al. examined muscle SNA in nine patients already fitted with a continuous flow LVAD (Heartwear). Incremental pump support which led to reductions in pulse pressure was associated with reductions in muscle SNA such that higher continuous flow LVAD support was shown to reduce muscle SNA (Tank et al., 2012).

Markham et al. compared MSNA in patients with continuous (*n* = 11) versus pulsatile LVADs (*n* = 6) at supine and head‐up‐tilt positions. Continuous flow LVAD patients exhibited significantly elevated muscle SNA burst frequencies in supine and head‐up tilt positions compared to the pulsatile flow LVAD group and healthy controls (*n* = 9). However, no significant differences in muscle SNA existed between the pulsatile flow LVAD patients and healthy controls in either position (Markham et al., 2013). Interestingly, Tank et al. conducted a similar head up tilt experiment but found no significant differences in muscle SNA between continuous and pulsatile flow LVADs regardless of positioning (Tank et al., 2012).

**FIGURE 2:**
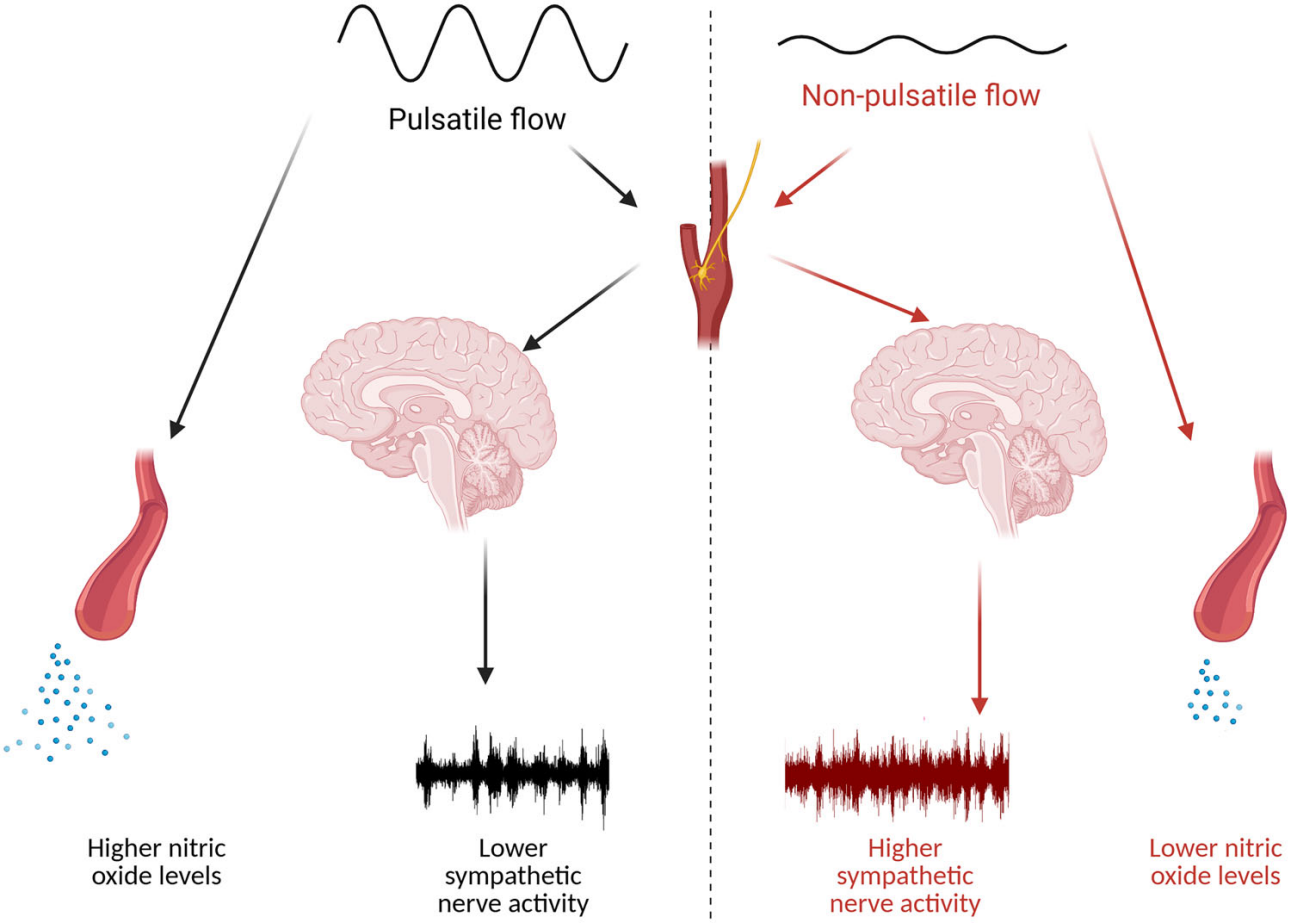
Effects of pulsatile flow on sympathetic nerve activity and nitric oxide release. Schematic representation indicating that pulsatile flow can increase production of nitric oxide from the endothelium, thereby reducing vascular resistance compared to continuous flow. In addition, non‐pulsatile or continuous flow tends to alter baroreflex sensitivity leading to higher levels of sympathetic nerve activity.

Cornwell et al. (2015) did not compare continuous flow LVAD results to those with pulsatile flow devices but instead compared a continuous flow LVAD at higher rotational speeds with lower rotational speeds. The lower rotational speed incorporates more physiologically normal pulsatility in blood flow compared to the high rotational speed continuous flow (Patel & Jorde, 2016). The study reported that muscle SNA was significantly reduced in continuous flow LVAD patients with lower rotational speeds (and thus with increased pulsatility) compared to those operating at higher rotational speeds. Furthermore, the change in muscle SNA was most prominent when the pump speed was reduced from baseline to the lower speed (Cornwell et al., 2015). Similar to muscle SNA, noradrenaline levels in supine continuous flow LVAD patients were higher than the noradrenaline levels during pulsatile flow (Markham et al., 2013). Taken together, this study suggests that in patients with continuous flow LVADs, the baroreflex mediated sympatho‐inhibition is sub‐optimal compared to that with pulsatile flow LVADs (Figure 2). It is important to highlight that the majority of these studies tend to be prospective with limited sample sizes. Moreover, the device type that leads to continuous or pulsatile flow tends to be different in each study, which limits easy comparison across studies. As such, there is a need for better designed interventional studies to better understand how pulsatility may alter SNA, especially in the long‐term.

## 5 ENDOTHELIAL RESPONSES TO PULSATILE SHEAR STRESS THAT INFLUENCE NITRIC OXIDE BIOAVAILABILITY

The importance of NO in the cardiovascular system was reiterated by the award of the Nobel Prize in Physiology or Medicine in 1998 to Robert Furchgott, Louis Ignarro and Ferid Murad who discovered the effect of endothelial‐derived NO on vascular resistance. Relevant to this review, flow pulsatility was found to be very important in maintaining endothelial‐derived NO release. Using in vitro (Hendrickson et al., 1999; Hishikawa & Lüscher, 1997) and in vivo (Baba et al., 2004; Canty & Schwartz, 1994; Nakano et al., 2000, 2001; Recchia et al., 1996) animal studies it has been demonstrated that pulsatile flow induces production of NO from the endothelium, reduces vascular resistance and improves tissue perfusion compared to continuous flow (Figure 2). Indeed, higher frequency of pulse waves (Hutcheson & Griffith, 1991; Nakano et al., 2001) and higher pulse pressures increase plasma NO metabolite concentration (Nakano et al., 2001).

One way to investigate how pulsatility alters NO levels is the use of cardiopulmonary bypass, which allows direct modulation of pulsatility in humans. An elegant study compared both a beating heart to continuous flow cardiopulmonary bypass and a mechanical pulsatile cardiopulmonary bypass to continuous flow cardiopulmonary bypass in humans undergoing cardiac surgery. In this study, a higher level of nitric oxide (plasmatic NO_2_^−^ and NO*_x_* concentrations) was noted among those exposed to a pulsatile flow compared to continuous flow. These changes were observed in surgeries lasting for 1–3 h, indicating a quick endothelial response to changes in the flow pattern (Lanzarone et al., 2009). These data would suggest that the presence of pulsatility would both improve levels of SNA and enhance NO release (Figure 2), both of which should be beneficial in the setting of HF.

## 6 DOWNSTREAM REGULATION OF RENAL MICROVASCULAR PERFUSION

The studies reviewed so far suggest that continuous flow may be detrimental to end‐organ function compared to pulsatile flow. The kidneys receive ∼20% of total blood volume to support the roles of filtration, excretion and homeostasis (O'Connor et al., 2006). The kidneys have an autoregulatory capacity such that small reductions in arterial pressure are offset by renal vasodilation to ensure that renal tissue is protected from hypoxia. These autoregulatory mechanisms are driven by a myogenic response and tubulo‐glomerular feedback (Burke et al., 2014; Carlström et al., 2015). When circulatory support systems employ non‐physiological flow patterns, particularly continuous flow, understanding the implications of this flow pattern for renal function becomes essential.

Regarding the mechanisms of autoregulation, transient receptor potential vanilloid (TRPV) channels, particularly TRPV1 and TRPV4, serve as key mechanosensitive channels within the renal vasculature (Chen et al., 2015). These TRPV channels are expressed on both endothelial cells and smooth muscle cells. Activation of these channels following pressure or stretch causes influx of Ca^2+^ ions and can modulate vascular tone (Baylie & Brayden, 2011). The resultant changes in vascular tone in response to the shear stress contributes to renal blood flow autoregulation. TRPV1 channels, which are predominantly expressed in preglomerular arterioles (Chen et al., 2015), play a direct role in the myogenic response. An increase in circumferential stretch activates TRPV1 channels in vascular smooth muscle cells, resulting in vasoconstriction to buffer a rise in perfusion pressure (direct action) (Phan et al., 2022). Paradoxically, TRPV1 activation can also elicit vasodilation through endothelial NO production. Additionally, Ca^2+^‐induced activation of intermediate and small conductance Ca^2+^‐activated K^+^ channels (IK and SK channels) can further hyperpolarize the cells promoting vasodilation (Earley et al., 2009; Phan et al., 2022). In addition to TRPV1, activation of TRPV4 channels in both pre‐ and post‐glomerular vessels (Chen et al., 2015) promotes autoregulatory vasodilation (Baylie & Brayden, 2011). Their activation leads to Ca^2+^‐induced Ca^2+^ release from sarcoplasmic stores via activation of ryanodine receptors. This elevated local Ca^2^^+^ induces large‐conductance Ca^2^^+^‐activated K^+^ channels (BK channels) to open. The resultant hyperpolarisation relaxes the smooth muscle cells to promote vasodilation (Earley et al., 2005).

Beyond the direct vascular effects, TRPV1 can also function as a mechanochemical receptor within renal afferent nerves and can alter efferent sympathetic drive and kidney function through the reno‐renal reflex. Indeed, activation of TRPV1 by increased renal pelvic pressure, ischaemia, inflammatory mediators and tubular fluid composition has been shown to activate renal sensory nerves. The enhanced afferent nerve activity can reduce efferent sympathetic nerve activity, promoting renal vasodilation, natriuresis and diuresis (Stocker & Sullivan, 2023). In chronic kidney disease, this inhibitory reflex can be attenuated thereby causing sympathoexcitation (Rahman et al., 2025).

Whether mediated by direct effects or through afferent nerves, these mechanosensitive pathways are particularly responsive to pulsatile flow. Cyclical stretch of the endothelial and vascular smooth muscle cells during pulsatile flow can enhance the dynamic responsiveness of the renal vasculature. The enhanced autoregulatory responsiveness of the renal vasculature is thought to help maintain a stable glomerular filtration rate, underscoring the importance of pulsatility. However, direct experimental evidence on how altered pulsatility affects mechanosensitive channel function in peripheral circulatory beds remains limited.

### 6.1 Outcomes for renal function using glomerular filtration rate

The comparisons of patients using pulsatile and continuous flow LVADs have used different outcome variables. Usually, estimated glomerular filtration rate was the more commonly used mode of renal function measurement. Although pulsatile flow LVADs maintain the pulsatile nature of regular human physiology, a greater number of studies have examined the role of a continuous flow LVAD indicating that this flow pattern still benefits renal function post‐implantation. In a retrospective cohort study, Quader et al. (2020) reported that patients implanted with continuous flow LVADs (*n* = 47) significantly increased their estimated glomerular filtration rate at 1, 3 and 6 months compared to preoperative recordings, suggesting that LVADs, even with continuous flow, protect renal function. Hasin et al. (2012) demonstrated similar results in a retrospective study from 83 patients implanted with continuous flow LVADs. At 1, 3 and 6 months post‐LVAD implantation, there was a significant increase in estimated glomerular filtration rate compared to pre‐LVAD values. Kikuchi et al. (2019) recorded glomerular filtration rate from severe heart failure patients (*n* = 41) implanted with continuous flow LVADs in a retrospective observational cohort study. Compared to preoperative values, estimated glomerular filtration rate was significantly greater post‐implantation for up to 24 months. All these studies indicate that an LVAD, irrespective of flow type is still beneficial and this reflects the importance of the improvement in cardiac output and perfusion of the kidney leading to improved outcomes.

Regarding whether pulsatile flow is better, a retrospective analysis was done by Sandner et al. (2008). Compared to pre‐implantation, continuous flow (*n* = 63) and pulsatile flow (*n* = 29) patients with LVADs had significant improvements in estimated glomerular filtration rate, though there were no significant inter‐device type differences suggesting that pulsatility does not appear to cause an improvement. A similar finding was reported in a retrospective study in adult patients fitted with LVADs (*n* = 3363) which observed significant early improvement in estimated glomerular filtration rate amongst both flow types. Moreover, no significant differences were found when directly comparing flow types (Brisco et al., 2014). Bartfay et al. (2022) also found no significant difference in glomerular filtration rate between continuous versus pulsatile flow LVAD patients. These studies suggest that pulsatility may not matter for clinical outcomes at least in terms of glomerular filtration rate measurements. These studies suggest that the improvement in cardiac output with the LVAD may override any minor changes in flow type, at least when examining outcomes based on estimated glomerular filtration rate.

Another factor to consider is the time course of the improvement. In this context, Brisco et al. (2014) reported a deterioration in estimated glomerular filtration rate amongst both continuous and pulsatile flow LVAD patients following the early improvement phase. In support of this, Hasin et al. (2012) also showed that estimated glomerular filtration rate declined to approximately preoperative values following continuous flow LVADs for 6 months. Thus, while renal function as measured by glomerular filtration rate did improve initially following LVAD implantation, it began to decline in most patients after 6 months.

### 6.2 Outcomes for renal function using creatinine‐based measures

Compared to glomerular filtration rate, three retrospective studies evaluated kidney function using creatinine‐based markers. The consensus is that both continuous and pulsatile flow LVADs appear to have comparable effects on kidney function. Sajgalik et al. (2012) conducted a retrospective analysis of patients implanted with either continuous (*n* = 119) or pulsatile (*n* = 8) flow LVADs and assessed plasma creatinine levels from baseline (implantation) to 6 months post‐implantation. At baseline, plasma creatinine was higher in pulsatile flow LVAD recipients but decreased significantly by 3 months. Similarly, continuous flow LVAD patients showed reduced plasma creatinine at 3 months. From 3 to 6 months, both groups demonstrated a significant increase in plasma creatinine; however, there were no significant differences between flow types (Sajgalik et al., 2012). Kamdar et al. (2009) and Radovancevic et al. (2007) also performed retrospective analyses, using creatinine clearance as the primary marker of kidney function. Kamdar et al. reported a significant increase in creatinine clearance in both continuous (*n* = 40) and pulsatile (*n* = 18) flow LVAD groups within 3 months of implantation, with no significant differences between device types. In contrast, Radovancevic et al. observed no significant changes in creatinine clearance in either group (*n* = 70) over a 15‐month follow‐up period. Thus, while the impact of LVADs on creatinine markers was not uniform, all three studies concluded that there was no difference in kidney function dependent on flow type. In terms of trying to explain the difference between the glomerular function studies and the creatinine studies, the limitations of using creatinine‐based markers needs to be considered. Among patients with HF warranting management with LVADs, alterations in muscle mass and volume status can impact creatinine levels. Ideally, alternative filtration markers such as cystatin C and biomarkers of tubular injury and function, such as neutrophil gelatinase‐associated lipocalin and kidney injury molecule‐1 are more appropriate (Larstorp et al., 2022; Mullens et al., 2020). There is a clear need for more studies that have used more robust markers of renal function post LVAD use.

### 6.3 Outcomes investigating distribution of blood flow in the kidney

While creatinine and glomerular filtration rate may not change, other studies have investigated whether pulsatility alters distribution of blood flow within the kidney. Interestingly, when more specific microcirculation markers were used, there appeared to be a difference with the flow type used. Sezai et al. (1997) examined the renal microcirculation in pig models (*n* = 6) with induced acute myocardial infarction and resulting cardiogenic shock. Immediately post‐cardiogenic shock, blood flow to the renal cortex decreased. Following assist device implantation, the group with pulsatile flow showed significantly increased microcirculation to the renal cortex after 6 h of support. In contrast, flow to the cortical microcirculation decreased significantly in the continuous group. These changes appeared to be region specific. At the renal medulla, flow to the microcirculation increased post‐cardiogenic shock in both groups, which seemed counter‐intuitive. Moreover, there was a reduction post‐LVAD implantation in both groups, with a greater reduction in the continuous group, who had a rapid decline in medullary circulation after 3 h of support. From 4 h onwards, there was a significant difference in microcirculation between flow types, with the continuous flow group being lower (Sezai et al., 1997).

Orime et al. (1996) similarly used pig models that were implanted with continuous (*n* = 7) or pulsatile (*n* = 7) flow LVADs following an acute myocardial infarction and cardiogenic shock. These studies indicated reduced renal cortical blood flow immediately post‐shock. Following assist device implantation, the pulsatile flow group had a significant increase in cortical microcirculation, whereas the continuous flow group demonstrated no significant change in blood flow. Following cardiogenic shock, there were no significant differences in renal medulla microcirculation in either group (Orime et al., 1996) contrary to the findings of Sezai et al. Taken together these findings suggest that pulsatile flow may result in beneficial improvements in regional blood flow distribution in the kidney.

The incidence of acute kidney injury has also been taken as a measure of nephrological clinical outcomes. Some studies report a lower acute kidney injury incidence among patients on pulsatile flow during cardiopulmonary bypass (Sevinc et al., 2019; Ueda et al., 2025). However other studies show no significant difference in the incidence of post‐operative acute kidney injury between the two modes of flow (Coulson et al., 2020). A meta‐analysis reported a higher incidence of acute kidney injury with non‐pulsatile perfusion after cardiac surgery, although renal failure prevalence was similar between the two groups, leading the authors to conclude that preserving pulsatility is acutely reno‐protective (Nam et al., 2015). Pulsatile flow has been associated with improved metabolic markers in some studies. Among patients undergoing cardiac surgery with cardiopulmonary bypass, higher urine output and lower serum creatinine levels were reported with pulsatile flow (Kocakulak et al., 2005). However, the need for colloids in the pulsatile group was significantly higher (to maintain pulsatility), potentially influencing the improvement in renal parameters. In a group of patients with comparable renal function at baseline, markers of renal injury (urinary neutrophil gelatinase‐associated lipocalin and interleukin 18) during cardiopulmonary bypass were lower among those perfused with pulsatile flow during the procedure (Adademir et al., 2012). More relevant to the current review, assessment of renal function among 92 patients with LVADs did not show a difference in renal function or adverse renal outcomes between pulsatile (*n* = 29) and continuous flow LVADs (*n* = 63) (Sandner et al., 2008). One important consideration in all of the cardiopulmonary bypass studies is that pulsatile flow can be generated by the pulse mode function on the arterial roller pump. This does not necessarily restore pulsatility to the same extent as other devices. These findings further highlight the context‐dependent nature of flow effects on renal outcomes. Taken together, studies indicate that pulsatile flow LVADs may alter renal microcirculatory flow in subtly different ways to continuous flow LVADs.

### 6.4 Insights from other vascular beds investigating microcirculation changes with altered pulsatility

We have focussed on the alterations in renal outcomes with pulsatility changes, but previous studies have examined the microcirculation in other vascular beds as well. In this context, Walshe et al. (2011) assessed the importance of pulsatility in regulating microvascular function in the retina within bovine models. Pulsatile flow promoted the survival of retinal endothelial cells and enhanced bovine retinal pericyte apoptosis by activating endothelial‐derived vasoactive substances, such as NO indicating a positive effect of pulsatility. In agreement with this, O'Neil et al. (2018) conducted a cohort study with patients who were put on either pulsatile (*n* = 10) or non‐pulsatile (*n* = 10) cardiopulmonary bypass during cardiac surgery. Sublingual mucosal microcirculation was examined, and pulsatile cardiopulmonary bypass maintained homeostatic perfusion characteristics, whereas perfusion deteriorated in the non‐pulsatile cardiopulmonary bypass cohort. More importantly, the study reported improved microcirculatory responsiveness during pulsatile cardiopulmonary bypass compared with non‐pulsatile cardiopulmonary bypass. The pulsatility in cardiopulmonary bypass is therefore likely to play a role in improving microcirculatory blood flow at the sublingual mucosal microvascular bed (O'Neil et al., 2018). In contrast to the previous two studies, Rossi et al. (2025) examined endothelial function in retinal arteries after ventricular assist device implantation in advanced heart failure patients. While endothelial function of larger arteries in the retina was significantly better in patients after assist device implantation, there was no difference in vascular function between patients with continuous (*n* = 26) versus pulsatile (*n* = 8) assist device implantation although the sample size is low in the pulsatile group (Rossi et al., 2025). Cornwell et al. (2019) monitored brain microvascular blood flow and cerebral perfusion in rabbit models during cardiopulmonary bypass and found brain blood flow was preserved regardless of exposure to non‐physiological pulse with minimal to no pulsatility. Taken together, the consensus seems to be that pulsatility may be important in regulating microvascular function, notably at the retina and sublingual mucosa.

## 7 DISCREPANCY BETWEEN PHYSIOLOGICAL STUDIES AND CLINICAL OUTCOMES

A general theme in the literature is the lack of agreement between the physiological effects of pulsatility (SNA and NO release) versus the lack of an effect in clinical studies (estimated glomerular filtration rate or creatinine measures). There are several reasons that may help explain the discrepancy. First, it is difficult to adequately control for varying cardiac output as a dependent variable in clinical scenarios. LVAD support clearly improves renal outcomes compared to no support. This improvement in cardiac output may override any putative additional benefits of pulsatility. In the clinical setting this is further complicated with the addition of other medications (antihypertensives and heart failure medications) which may improve outcomes to a greater extent than improvements with pulsatility. Second, the experimental studies suggest pulsatility may alter both sympathetic drive and the redistribution of blood flow within the kidney. It is possible that this redistribution of blood flow within the kidney may over‐ride any putative effects on sympathetic outflow. Therefore, the resultant outcomes in kidney function measured using either glomerular filtration rate or creatinine clearance are not different. Finally, it is worth noting that once the LVAD restores perfusion pressure, even if pulsatility alters sympathetic drive to the kidney, renal autoregulation may ensure that there are no differences between the two modes of perfusion.

## 8 CONCLUSION

In the clinical context of ventricular dysfunction and mechanical circulatory support, experimental studies have observed that SNA levels are lower with pulsatile flow and should theoretically improve NO function. The effect of pulsatile flow on renal function in patients, however, remains inconclusive with the caveat that these studies are often underpowered retrospective observational studies that typically use indirect measures of renal function. Studies that have examined microcirculatory changes in the kidney and other organs have suggested that pulsatility can lead to differential redistribution of blood flow. The lack of an effect on renal function may be explained by the improvement in cardiac output and redistribution of blood flow within the kidney with both types of LVAD support. These effects may override any putative beneficial effects of altered pulsatility on SNA and NO release. In addition, the limitations of using creatinine‐based markers in clinical studies needs to be considered. Future studies need to incorporate more sensitive markers of tubular and glomerular function to determine if the SNA and microcirculatory changes with pulsatile flow observed in experimental studies can lead to meaningful positive outcomes in patients.

## AUTHOR CONTRIBUTIONS

Rohit Ramchandra and Tania Warnakulasuriya conceived the review topic. Tania Warnakulasuriya and Zaid Bahi performed the literature search, synthesized the evidence, drafted the initial manuscript, and Rohit Ramchandra critically revised the manuscript. All authors have read and approved the final version of this manuscript and agree to be accountable for all aspects of the work in ensuring that questions related to the accuracy or integrity of any part of the work are appropriately investigated and resolved. All persons designated as authors qualify for authorship, and all those who qualify for authorship are listed.

## CONFLICT OF INTEREST

None declared.
